# Impact of a Preemptive Multimodal Analgesia plus Femoral Nerve Blockade Protocol on Rehabilitation, Hospital Length of Stay, and Postoperative Analgesia after Primary Total Knee Arthroplasty: A Controlled Clinical Pilot Study

**DOI:** 10.1100/2012/273821

**Published:** 2012-04-30

**Authors:** Lauren A. Beaupre, D. Bill C. Johnston, Sherry Dieleman, Ban Tsui

**Affiliations:** ^1^Department of Physical Therapy, University of Alberta, Edmonton, AB, Canada T6G 2G4; ^2^Division of Orthopaedic Surgery, Department of Surgery, University of Alberta, 1F1.52 WMC, 8440-112 Street, Edmonton, AB, Canada T6G 2B7; ^3^Department of Anesthesiology and Pain Medicine, Stollery Children's Hospital, University of Alberta, Edmonton, AB, Canada T6G 2B7

## Abstract

*Purpose*. To compare preemptive multimodal analgesia (PMMA) without femoral nerve blocks (FNB) to PMMA including FNB following total knee arthroplasty (TKA). *Methods*. In a prospective, controlled pilot study, subjects with noninflammatory arthritis undergoing TKA and a short postoperative stay received either PMMA + FNB (FNB group; *n* = 19) or PMMA only (PMMA group; *n* = 20). No preoperative group differences were noted. Evaluations occurred in hospital and at 2, 6, and 12 weeks postoperatively. The primary outcome (knee flexion) was measured on day two postoperatively. Rehabilitation indices, pain, analgesic use, and length of stay (LOS) were also measured. *Results*. All subjects completed the study. The only significant group differences were quadriceps motor blocks in the FNB group (*P* < 0.001). No significant differences were noted in ROM, pain levels, analgesic use, or hospital LOS. *Conclusion*. Other than the quadriceps motor block, no group differences were noted; both achieved satisfactory analgesia. Best postoperative pain management strategies when following a short hospital stay program are still unclear.

## 1. Introduction

Total knee arthroplasty (TKA) is a common procedure for treatment of end-stage arthritis and can significantly improve pain and function [1]. Despite long-term benefits, TKA is also associated with substantial early postoperative pain; thus, pain management is critical [2]. 

During the past decade, perioperative care for patients undergoing TKA has been changing rapidly including analgesia. In the 1990s, patients frequently remained in hospital for more than 1 week postoperatively [3, 4]. This is in direct contrast to current reports of hospital length of stay (LOS) of three days or less [5, 6]. To achieve early discharge, rehabilitation is often started the day of surgery. Thus, optimal pain management is vital to allow patients to tolerate early and frequent therapy during their short hospital stay. 

Accompanying changes in postoperative care are multiple pain management strategies including multimodal analgesia, nerve blockades, and preemptive analgesia [2, 7–9]. Multimodal analgesia regimens often combine oral medications (e.g., nonnarcotic analgesics, narcotic analgesics, and anti-inflammatory medication) and nerve blockades to decrease narcotic consumption. 

Femoral nerve blockades (FNBs) significantly improve pain and knee range of motion (ROM) and reduce narcotic consumption relative to patient-controlled analgesia (PCA) or oral/intravenous narcotics [8, 10–13]. However, FNBs are also frequently associated with quadriceps weakness and have been associated with falls postoperatively [14]. Thus, their overall effectiveness is uncertain when early discharge programs are utilized [15, 16]. 

Preemptive multimodal analgesia (PMMA) is also being used where patients take analgesics preoperatively (preemptive) to prevent central sensitization and amplification of postoperative pain and combine multiple types/routes of analgesics (multimodal) to maximize analgesia with the fewest side effects [2, 9, 17, 18]. PMMA often includes FNB; there appear to be few reports of PMMA without FNB. 

This pilot study examined the impact of the PMMA regimen with the addition of an FNB compared with usual care—an oral PMMA regimen without FNB. The overall goal was to examine the effect of PMMA including FNB on discharge knee flexion and in-hospital pain compared to PMMA without FNB following primary TKA. Further, we compared the impact of the pain management strategies on (a) rehabilitation indices, (b) hospital LOS, (c) analgesic use, and (d) pain and knee ROM at two, six, and 12 weeks postoperatively. 

The primary objective was to determine the impact of PMMA with FNB on knee flexion measured on day two postoperatively compared to PMMA without FNB. Secondary objectives were to compare postoperative pain, rehabilitation indices (quadriceps block and rehabilitation participation) during the first two postoperative days, and hospital LOS in these same two patient groups. Finally, we compared postoperative analgesic use and postdischarge pain and knee ROM at weeks two, six, and 12 postoperatively.

## 2. Materials and Methods

This was a prospective pilot study undertaken at two tertiary hospitals that perform high volumes of TKA annually. Both hospitals followed the same regional clinical pathway for preoperative, perioperative, and postoperative care, including standardized medical and analgesic orders as well as a standardized rehabilitation approach for postoperative care. Care standardization is evaluated across sites to ensure that sites are compliant with the regional clinical pathway.

All subjects (*n* = 39) were managed at the same central intake and follow-up clinic that used standardized pre- and posthospital care. This sample size allowed detection of 10-degree difference in knee flexion (*α* = 0.05; power = 0.80), while accounting for 10% attrition, appropriate for our pilot study.

In one hospital, anesthesiologists added FNB to the PMMA regimen (FNB group (*n* = 19)), while the second site used PMMA without FNB (PMMA group (*n* = 20)) as per usual care. Oral medications dosages were adjusted in the FNB group to ensure adequate pain control as needed. All subjects provided informed consent, and the Health Research Ethics Board approved the study.

To be eligible, subjects had preoperative knee ROM ≥90 degrees, body mass index (BMI) less than 40, and no known hepatic insufficiency or contraindications to FNB and did not regularly use narcotics preoperatively. Subjects were English-speaking so that pain could be accurately assessed using a visual analogue scale (VAS).

Subjects who met the selection criteria and were willing to participate signed an informed consent preoperatively. Subjects reported current pain levels using a VAS, preoperative analgesic use (both narcotic and nonnarcotic), activity levels, and general demographic/medical (e.g., age, sex, and comorbidities) information to a research associate not involved in their clinical care. Knee ROM was also assessed.

All surgeries were done using standard surgical technique (i.e., midline incision and medial parapatellar exposure). No minimally invasive techniques were used. In the FNB group, 17 (89%) subjects received general anaesthesia with the others (11%) receiving spinal anaesthesia. In the PMMA group, 16 (80%) received spinal anaesthesia with the remaining subjects undergoing general anaesthesia.

### 2.1. Preemptive Analgesia

All subjects in both groups received Oxycodone controlled release (10 milligrams (mg) per oral (PO)) and Celecoxib (100 mg PO) within two hours of surgery. Celecoxib (100–200 mg PO) was administered twice daily starting 12 hours after the preoperative dose and continued throughout their hospital stay for all subjects.

### 2.2. PMMA

Postoperatively, the PMMA group received Oxycodone controlled release (10–20 mg PO) twice daily for five doses starting 12 hours after the preoperative dose. For breakthrough pain, subjects received up to two tablets of acetaminophen (325 mg with codeine 30 mg) or acetaminophen (325 mg with oxycodone 5 mg) every four hours as required. These were the primary analgesics used once the Oxycodone controlled release doses were completed. If oral analgesics were ineffective, subcutaneous morphine or hydromorphone (if intolerant of morphine) was used with a dose range of 0.5–3 mg.

### 2.3. FNB

FNB subjects had femoral nerve catheters placed preoperatively using ultrasound guidance via in-plane approach. Choice of concentration, type, and volume of local anesthetic was left to the attending anesthesiologist's discretion. A local anesthetic bolus of 0.1–0.5% ropivacaine was administered via the femoral catheter with or without 0.125–0.25% bupivacaine (median 20 millilitres (mL), 0.25% ropivacaine, and 0.125% bupivacaine). In addition, 16 (84%) subjects also received a single-shot anterior sciatic nerve block at the time of surgery at the discretion of the attending anesthesiologist. Subjects were then anesthetized for the TKA in the operating room. Subjects received narcotics intraoperatively at the attending anesthesiologist's discretion. All morphine received was recorded. 

Subjects were monitored regularly while the FNB was in situ receiving 0.1-0.2% ropivacaine at a rate of 1–6 mL per hour as needed for pain control. Subjects with continuous infusion received an additional 4–8 mL per hour while those receiving intermittent boluses (2% lidocaine, 0.5% bupivacaine, and/or 0.1-0.2% ropivacaine) had varying quantities (6–30 mL). Anesthesiologists ordered additional analgesia as required for adequate analgesia following the recommendations of the oral PMMA regimen. The FNB was discharged on the second postoperative morning after which subjects followed the PMMA regimen until hospital discharge. All narcotic use was recorded.

### 2.4. Postoperative Management

Mobilization started the day of surgery at both sites as all subjects followed the same regional pathway; subjects sat on the edge of the bed or in a chair, or ambulated up to five feet, dependent upon their tolerance. On each subsequent day, subjects attended rehabilitation twice a day to perform exercises and ambulate. The goal was discharge home on the third postoperative morning. To be discharged, subjects had adequate pain control on oral analgesics, performed self-care and transfers independently, and ambulated weight-bearing as tolerated independently with walking aids. Although not necessary for discharge, the goal was at least 60 degrees of knee flexion by the second postoperative day.

Knee flexion and pain were measured daily in hospital. We expected to measure knee flexion at hospital discharge, but subjects were frequently discharged on day three prior to measuring ROM. Thus, to fairly compare postoperative knee flexion between groups, the primary flexion comparison occurred on day two postoperatively.

Rehabilitation indices (quadriceps block, participation in rehabilitation), hospital narcotic analgesic use, and LOS were also recorded. After discharge, pain and knee flexion were evaluated at two, six, and 12 weeks postoperatively.

## 3. Outcome Measures

### 3.1. Knee Flexion

Knee flexion was measured in supine lying using a goniometer, which has been shown to be reliable [19].

### 3.2. Pain

Pain was measured at the start of rehabilitation using an 11-point VAS (0–10), shown to be a reliable and valid method of measuring patient-reported pain [20]. Subjects gave their verbal pain rating based on zero being “no pain present” and 10 being “the worst pain imaginable.”

### 3.3. Quadriceps Block

Subjects had a positive quadriceps block if they were unable to actively contract or produce a motor response of their quadriceps.

### 3.4. Participation in Rehabilitation

Participation in rehabilitation was measured as a count of rehabilitation sessions. Subjects were expected to have one session on the day of surgery and two sessions/day thereafter in hospital.

### 3.5. Analgesic Use

All narcotic medication was recorded and standardized. For the first three postoperative days (including surgical day), the total amount of any narcotic used was converted to the equivalent IM morphine dose using a morphine equivalency scale [21]. The resulting equivalents were totaled to give a single score reflecting the total narcotics used. 

## 4. Analysis

Knee flexion at discharge was the primary outcome and was analyzed using independent *t*-tests. Because there were no baseline differences noted between groups, the mean postoperative knee flexion was chosen as the appropriate comparison. Pain levels were also assessed using independent *t*-tests. Rehabilitation indices (quadriceps block and rehabilitation participation) and analgesic use were analyzed using chi-square tests. LOS was analyzed using the Mann-Whitney *U* test for nonparametric data. Postdischarge pain and knee flexion were analyzed using independent *t*-tests.

Statistical analyses were performed with Predictive Analytics SoftWare (PASW) version 17.0 (SPSS: An IBM Company, Chicago, Ill, USA) utilizing 2-tailed tests. Although we calculated the sample size based on a level of significance of *P* < 0.05, we reset the level of significance at *P* < 0.01 prior to analysis because we evaluated multiple outcomes over repeated time intervals.

## 5. Results

### 5.1. Baseline Characteristics

Subjects were similar in age, gender distribution, and comorbidities and reported no differences in preoperative function and knee flexion (Table 1). All subjects completed the study. Use of preoperatively analgesic medication was also similar between groups (*P* > 0.80) although the FNB group reported nonsignificantly higher pain levels preoperatively than the PMMA group (Table 1).

### 5.2. Knee Flexion

Knee flexion was similar between groups on both the first and second postoperative days (Table 2).

### 5.3. In Hospital Postoperative Pain and Analgesic Use

The FNB group reported nonsignificantly higher mean pain scores during the hospital stay (Table 2). In terms of medication use, the median morphine equivalency units for in-hospital analgesic use were nonsignificantly lower in the FNB group (59.6 (IQR 46.6, 130.4) versus 67.4 (IQR 46.4, 84.3)) than in the PMMA group (*P* = 0.19 by Mann Whitney *U* test), but the mean analgesic use was nonsignificantly higher in the FNB (84.17 (51.2)) than in the PMMA (67.33 (24.3)) group (*P* = 0.21 by independent *t*-test).

### 5.4. Rehabilitation Indices

FNB subjects were significantly more likely to have quadriceps blocks on the day of surgery (day 0) and first postoperative day than the PMMA subjects (Table 2). Fewer FNB subjects were able to participate in rehabilitation on the day of surgery, but this difference was not significant (*P* = 0.32). Despite more quadriceps blocks in the FNB group, no subjects in either group missed rehabilitation sessions after the day of surgery. Further, no subjects experienced any falls postoperatively.

### 5.5. LOS

The median LOS was 4 (interquartile range 4,5) days in the FNB group and 3.5 (3,4) days in the PMMA group (*P* = 0.04), although this difference did not reach the *a priori* level of significance, which was set at *α* = 0.01.

### 5.6. Postdischarge Pain and Knee Flexion

There was no difference in pain or knee ROM at the postdischarge follow-ups (Table 3). Few subjects in either group were using narcotic analgesic beyond the two-week postoperative visit with similar narcotic use between groups (*P* > 0.42).

## 6. Discussion

Pain management following TKA is highly variable and continues to evolve with changing postoperative care. FNB appears more effective than PCA in managing postoperative pain, preventing nausea/vomiting, and increasing knee ROM following primary TKA [2, 8]. However, FNB has been associated with increased postoperative falls due to quadriceps weakness; thus their utility may be more limited with aggressive postoperative rehabilitation [14]. Our controlled pilot study compared two pain management protocols, one in which subjects followed an oral PMMA only and one in which subjects followed an adapted PMMA augmented by an FNB. We found no significant differences between groups in terms of knee flexion or reported postoperative pain up to 12 weeks postoperatively. Further, analgesic use did not appear different between groups. The FNB group reported more quadriceps motor blocks, a common side effect attributed to FNB [15, 16, 22, 23]. However, no patient experienced falls, and the two groups reported similar rehabilitation attendance in hospital. The FNB group stayed a half day longer in hospital than the PMMA group, but this difference was not significant. 

Past studies compared FNB to PCA regimens or to different nerve block techniques and demonstrated that FNB is an effective pain management strategy following TKA [10–13, 22, 24]. Recent guidelines suggest that FNB should be used as routine care following TKA [2]. However, based on the reported association of increased fall risk with FNB [14], further evaluation of FNB is necessary, particularly in the context of short stay postoperative protocols. Despite more quadriceps block in the FNB group, we found no difference in rehabilitation attendance or knee flexion between PMMA with FNB and PMMA alone; both groups reported similar postoperative analgesia. As previous reports on PMMA effectiveness have typically included FNB as part of the regimen [9, 24, 25], our study adds to current evidence by comparing the impact of PMMA with and without an FNB when a short stay postoperative protocol is utilized. 

Our study has several strengths; this was a prospective controlled pilot study where all participants were screened and followed postoperatively at a single centre and received standardized pre-, peri-, and postoperative care except for pain management. We also assessed several important outcomes, rather than just the most commonly reported pain or analgesia [10–12]. We examined the impact of the analgesic regimes on rehabilitation including knee flexion and quadriceps weakness. With evolving postoperative care moving to early rehabilitation and short LOS, patients' pain must be adequately controlled without impeding time to discharge or affecting patient safety. Therefore, these additional outcomes are important to measure to determine optimal pain management after TKA.

However, there are also limitations to our study. We did not randomize subjects but instead evaluated the impact of adding an FNB to an established oral PMMA regimen at one hospital. Thus, it is possible that the measured outcomes were affected by hospital differences despite using a regional care pathway. Further, FNB technique was not standardized and was left to the discretion of the attending anaesthesiologist. These variations could affect the FNB efficacy; however, our study reflects the effectiveness of an FNB applied under usual clinical standards as determined by the attending anaesthesiologist.

Finally, our pilot study is small; the study was under-powered for the primary outcome at the level of significance analyzed (*P* < 0.01). However, at no time was more than a six-degree difference in knee ROM reported between groups, so no clinically important differences were deemed nonsignificant by our revised level of significance. Further study is needed to determine if similar results occur using a randomized study design, standardized regimens, and a larger sample. 

There is much heterogeneity in regard to optimum post-TKA analgesia due to multiple changes in surgical practice and postoperative care, reducing the applicability of previous studies to current care. To date, there is no answer as to best practices for pain management following TKA. An oral PMMA regimen with or without augmentation of an FNB appears to provide adequate analgesia. Further research should also consider outcomes beyond analgesia and measure the impact of the analgesic regimen on patients overall postsurgical recovery. 

## Figures and Tables

**Table 1:** Baseline characteristics.

	FNB (*n* = 19)	PMMA (*n* = 20)	*P*-value
Demographics			
Male (%)	12 (57)	9 (43)	0.34*
Mean age (SD)	66.7 (7.9)	65.8 (8.4)	0.27^†^

Function			
Mean knee flexion (SD)	119.3 (11.5)	118.6 (10.9)	0.83^†^
Walks <6 blocks (%)	17 (91)	16 (80)	0.63*
Uses no aide (%)	10 (53)	13 (65)	0.33*

Pain			
VAS score (SD)	6.5 (2.3)	5.2 (2.3)	0.09^†^

FNB: femoral nerve block, PMMA: preemptive multimodal analgesia, SD: standard deviation, and VAS: visual analogue scale.

*Analyzed using chi-square test.

^†^Analyzed using independent* t*-test.

**Table 2:** In-hospital measures.

	FNB (*n* = 19)	PMMA (*n* = 20)	*P* value
Mean knee flexion			
Day 1 postoperatively (SD)	56.7 (16.8)	51.9 (11.3)	0.31^†^
Day 2 postoperatively (SD)	63.1 (16.2)	61.1 (12.1)	0.67^†^

Mean pain levels using VAS			
Day 1 postoperatively (SD)	5.0 (1.9)	4.3 (1.7)	0.21^†^
Day 2 postoperatively (SD)	5.4 (2.4)	4.1 (1.7)	0.08^†^

Rehabilitation indices			
Quadriceps block			
Day 0 (%)	10 (53)	0 (0)	<0.001*
Day 1 (%)	13 (68)	0 (0)	<0.001*
Day 2 (%)	4 (22)	0 (0)	0.046*

Missed rehabilitation participation			
Day 0 (%)	8 (42)	5 (25)	0.32*

FNB: femoral nerve block, MMA: preemptive multimodal analgesia, SD: standard deviation, and VAS: visual analogue scale.

^†^Analyzed using independent* t*-test.

*Analyzed using Chi Square test.

**Table 3:** Postdischarge outcomes.

	FNB	PMMA	*P* value
Postdischarge pain			
2 weeks (SD)	4.0 (2.6)	4.4 (2.4)	0.72^†^
6 weeks (SD)	2.8 (2.2)	2.9 (2.4)	0.93^†^
12 weeks (SD)	1.1 (1.9)	2.0 (2.2)	0.31^†^

Postdischarge knee flexion			
2 weeks (SD)	84.7 (10.4)	83.1 (9.8)	0.69^†^
6 weeks (SD)	101.3 (13.4)	97.4 (13.2)	0.40^†^
12 weeks (SD)	110.9 (9.6)	105.0 (11.7)	0.14^†^

FNB: femoral nerve block, PMMA: preemptive multimodal analgesia, SD: standard deviation, and VAS: visual analogue scale.

^†^Analyzed using independent* t*-test.
